# A Novel Focal Phi Loss for Power Line Segmentation with Auxiliary Classifier U-Net

**DOI:** 10.3390/s21082803

**Published:** 2021-04-16

**Authors:** Rabeea Jaffari, Manzoor Ahmed Hashmani, Constantino Carlos Reyes-Aldasoro

**Affiliations:** 1 Department of Computer and Information Sciences, Universiti Teknologi PETRONAS (UTP), Seri Iskandar 32610, Malaysia; manzoor.hashmani@utp.edu.my; 2 High Performance Cloud Computing Centre (HPC3), Universiti Teknologi PETRONAS (UTP), Seri Iskandar 32610, Malaysia; 3 giCentre, Department of Computer Science, University of London, London EC1V 0HB, UK; constantino-carlos.reyes-aldasoro@city.ac.uk

**Keywords:** power lines, semantic segmentation, Matthews correlation coefficient, loss function, data imbalance

## Abstract

The segmentation of power lines (PLs) from aerial images is a crucial task for the safe navigation of unmanned aerial vehicles (UAVs) operating at low altitudes. Despite the advances in deep learning-based approaches for PL segmentation, these models are still vulnerable to the class imbalance present in the data. The PLs occupy only a minimal portion (1–5%) of the aerial images as compared to the background region (95–99%). Generally, this class imbalance problem is addressed via the use of PL-specific detectors in conjunction with the popular class balanced cross entropy (BBCE) loss function. However, these PL-specific detectors do not work outside their application areas and a BBCE loss requires hyperparameter tuning for class-wise weights, which is not trivial. Moreover, the BBCE loss results in low dice scores and precision values and thus, fails to achieve an optimal trade-off between dice scores, model accuracy, and precision–recall values. In this work, we propose a generalized focal loss function based on the Matthews correlation coefficient (MCC) or the Phi coefficient to address the class imbalance problem in PL segmentation while utilizing a generic deep segmentation architecture. We evaluate our loss function by improving the vanilla U-Net model with an additional convolutional auxiliary classifier head (ACU-Net) for better learning and faster model convergence. The evaluation of two PL datasets, namely the Mendeley Power Line Dataset and the Power Line Dataset of Urban Scenes (PLDU), where PLs occupy around 1% and 2% of the aerial images area, respectively, reveal that our proposed loss function outperforms the popular BBCE loss by 16% in PL dice scores on both the datasets, 19% in precision and false detection rate (FDR) values for the Mendeley PL dataset and 15% in precision and FDR values for the PLDU with a minor degradation in the accuracy and recall values. Moreover, our proposed ACU-Net outperforms the baseline vanilla U-Net for the characteristic evaluation parameters in the range of 1–10% for both the PL datasets. Thus, our proposed loss function with ACU-Net achieves an optimal trade-off for the characteristic evaluation parameters without any bells and whistles. Our code is available at Github.

## 1. Introduction

Unmanned aerial vehicles (UAVs) with vision systems are usually employed for the automated inspection and maintenance of electrical power lines from aerial images. As compared to the manual inspection approaches, automated inspection and maintenance is more efficient because it is effortless, transient, easily accessible in remote areas and yields consistent inspection results [1,2]. At the heart of such UAV-based inspection systems lie the power line (PL) detection modules, which detect and localize the power lines from aerial images effectively so that the UAVs can safely navigate around them during the automated inspection and maintenance operations.

PLs are regarded as one of the most dangerous obstacles for UAVs due to their weak visual appearances and pervasive existence [3]. Hence, PL detection is an extremely challenging task because the morphologically thin and long PLs only occupy a minimal portion of the overall aerial image and are therefore difficult to recognize with high accuracy. Moreover, various lighting conditions (foggy, bright, etc.) and cluttered backgrounds (leaves, branches and tiles) further add to the difficulty of the detection task at hand [1,4,5].

The recent deep learning-based semantic segmentation networks have been applied successfully to the PL detection task to detect the PLs at the pixel level [4,6,7,8]. These networks frame PL detection as a binary classification problem with the goal of effectively classifying the PL pixels against the background pixels. However, such networks are susceptible to the class imbalance problem because PLs are thin, slender and less compact in nature as compared to the overwhelmingly larger and compact background. PLs only account for 1–5% of the total image pixels while the rest of the pixels (95–99%) correspond to the background pixels. This class imbalance introduces a high bias towards the majority class (background in this case), making it difficult for the deep networks to classify the foreground class of interest, also known as the minority class (PL in this case) accurately [9]. Studies confirm that class imbalance is significantly detrimental to the performance of deep classifiers as it not only affects the model convergence during training but also the model’s generalization capability during testing [9,10].

Amongst the available methods to tackle the class imbalance problem in deep segmentation networks, algorithm-level methods, particularly the novel loss functions, are most commonly used because they do not affect the training times by altering the data distributions and require little to no tuning. For a detailed survey on the methods to handle class imbalance in deep learning classifiers, we direct the interested readers to Johnson, J.M. and Khoshgoftaar, T.M. [11]. The loss functions shift the focus to the minority class, thus, allowing it to contribute more to the loss in comparison to the major background class. Despite the fact that class imbalance affects the PL detection task adversely, only a handful of PL detection works address the class imbalance problem either via the popular variant of the binary cross entropy (BCE) loss function, namely the balanced BCE (BBCE) loss [4,7,12], or via a compound loss function [8] or the attention gated networks [13]. However, other notable studies on PL detection do not consider the prevalent class imbalance problem and utilize the vanilla BCE loss function for training the deep semantic segmentation networks [6,14,15,16]. Moreover, BCE loss and its variants are also the usual choice for loss function in the PL detection tasks [4,6,7,12,14,15,16]. Nevertheless, it has been demonstrated that other kinds of losses can outperform the popular BCE loss for typical classification tasks [17,18]. Region-based losses such as the Dice loss (DL) [19], Tversky loss (TL) [20] and their variants [21,22,23], have outperformed the BBCE loss for handling the class imbalance issue in medical image segmentation [24] but these losses have not yet been extended to other semantic segmentation applications. Medical image segmentation is characterized via small and compact regions of interest (RoIs) and less severe class imbalance as compared to the thin RoIs and more severe class imbalance in PL detection. Hence, there is a need to thoroughly investigate the choice of loss function while considering the prevalent issue of class imbalance in PL detection so that the PLs with thin RoIs can be identified accurately.

In this research, we empirically investigate various loss functions to tackle the class imbalance and the subsequent thin RoIs identification problem in PL detection. We propose a novel focal loss function based on the Phi coefficient (also known as Matthews correlation coefficient, MCC) to optimize the deep segmentation network for PL detection. Our proposed loss function outperforms the existing baselines on two benchmark PL detection datasets by achieving an optimal trade-off for the evaluation parameters. We employ a U-Net [25]-based architecture as the baseline segmentation network for our empirical investigation due to its ability to handle vanishing gradients effectively and to incorporate multi-contextual feature information via skip connections.

### Contributions

The major contributions of this research are four-fold, namely:

1. A novel focal Phi loss (FPL) function for highly imbalanced data and thin RoI segmentation, where we modulate the Phi coefficient (also known as MCC) [26] to achieve an optimal trade-off between the evaluation parameters with minimal hyperparameter tuning.
2. Validation of the proposed novel FPL function in terms of characteristic evaluation parameters (accuracy, sensitivity, specificity, false detection rate (FDR), class-wise dice scores and precision) on two PL detection benchmark datasets with varying levels of class imbalance.
3. Empirical analysis of the choice of loss function for PL detection and evaluation of the proposed novel FPL function with state-of-the-art loss functions for handling class imbalance.
4. A deeply supervised U-Net, named the auxiliary classifier U-Net (ACU-Net), improved with the simple addition of a convolutional auxiliary classifier for faster model convergence and better feature representations.

The remainder of this paper is organized as follows. Section 2 of this study briefly discusses various loss functions for handling the class imbalance issue for semantic segmentation tasks in general and for PL detection in particular. Section 3 proposes the novel FPL function for highly imbalanced PL detection tasks and thin RoI segmentation along with the auxiliary classifier U-Net (ACU-Net). Section 4 presents the experimental results and discussions along with a detailed comparative analysis of the proposed FPL function and ACU-Net with established baselines. Finally, the conclusion and future work is presented in Section 5.

## 2. Related Work and Theoretical Foundation

A deep network tends to learn its desired objective, such as pixel classification, via the minimization of an appropriate loss function during training. The loss function is a mathematical formulation of the error between the expected results and the results predicted by the deep network. Several loss functions have been proposed in the literature to handle the class imbalance problem in deep semantic segmentation networks. These loss functions can be fairly divided into three categories: namely, the distribution-based loss functions, the region-based loss functions and the compound loss functions [27].

### 2.1. Distribution Based Loss Functions

Distribution-based loss functions stem from the distribution of class labels and tend to minimize the difference between the predicted and actual probabilities of image pixels during model training [27]. These include the BCE loss function (also known as the log loss) [28] and its class imbalance variants [29,30,31]. The BCE loss is defined as:

L_BCE_(y,ŷ) = −(y log (ŷ) + (1 − y) log (1 − ŷ)) (1)

Here, y and ŷ specify the actual and predicted probabilities, respectively. In the case of class imbalance, the BCE loss yields sub-optimal results which are biased towards the majority class because it weighs all pixels equally during the loss calculation. Hence, the deep network always receives a low loss, because the background pixels are classified easily, and the network does not learn the actual objective of classifying the minority class pixels accurately. This class imbalance is alleviated by assigning weights to either only the foreground class, i.e., the PLs in our study, as in the case of weighted BCE (WBCE) loss, or to both the classes in BBCE loss [29]. The WBCE and BBCE are presented in Equations (2) and (3), respectively.

L_WBCE_(y,ŷ) = −(β ∗ y log (ŷ) + (1 − y) log (1 − ŷ)) (2)

L_BBCE_(y,ŷ) = −(β ∗ y log (ŷ) + (1 − β) ∗ (1 − y) log (1 − ŷ)) (3)

The class weights (β and (1 − β)) are inversely proportional to the number of samples in the class. Another variant of the BCE loss that addresses the class imbalance problem is the focal loss (FL) [30] defined in Equation (4).

L_FOCAL_(p_t_) = − α_t_ (1 − p_t_) γ log (p_t_) (4)

Here, p_t_ is the predicted probability, α is the class weighting parameter while γ is the modulating factor that shifts the model’s focus on learning hard negatives during training by down weighting the easy (majority class) examples. 

To the best of authors’ knowledge, all the studies on PL detection utilize the BCE loss [6,14,15,16] and its class imbalance variants [4,7,12] for segmenting the PLs. Although BCE loss is easier to optimize with lower training times, it might not always be the best choice for training deep classification networks [17,18]. Moreover, BCE loss treats each pixel independently and does not consider the pixel level structural information and inter-pixel relationships [32]. Class imbalance variants of the BCE loss introduce additional weighting hyperparameters (α, β, γ) which need to be adjusted carefully. Lastly, experiments of the BBCE loss with benchmark PL detection datasets (Section 4.3.2) reveal that it achieves high accuracy and recall values but at the cost of dice scores and precision values. Thus, it fails to achieve an optimal trade-off between the accuracy, dice scores, precision, and recall values.

### 2.2. Region Based Loss Functions

Region-based or overlap metric-based loss functions directly measure the degree of overlap between the predicted and the actual image regions of the foreground class of interest only. Hence, region-based losses are usually preferred over BCE losses for handling skewed data because these losses reflect the segmentation quality in a better manner as compared to the BCE losses [24]. DL [19] based on the Sorenson–Dice index and TL [20] based on the Tversky index are the most commonly employed region-based losses for the semantic segmentation of skewed data. DL and TL are defined by Equations (5) and (6), respectively.

L_DICE_ = 1 − ((2TP + ε)/(2TP + FN + FP + ε)) (5)

L_TVERSKY_= 1 − ((TP + ε)/(TP + αFN + βFP + ε)) (6)

TP, FN, and FP signify the true positive, false negative and false positive predictions, respectively, and ε is the smoothing factor to prevent infinite values. α and β are the hyperparameters in TL (Equation (6)) which allow the FNs and FPs to be weighted differently as opposed to DL, which weighs both quantities equally (Equation (5)). TL simplifies to DL when α = β = 0.5. Although these weights improve the recall rate for skewed datasets, they require tuning to perform optimally. It should also be noted that none of these losses penalize the misclassifications of the background information, making it difficult to optimize them for accurate background predictions [26]. Moreover, these loss functions and their variants [21,22,23] have so far been applied to medical segmentation tasks only where the foreground/minority class is characterized by a small but compact RoI as opposed to PL detection tasks where the RoIs are thin and more widespread. Lastly, the class imbalance in PL datasets is more severe, i.e., 2:98 for the minority versus majority class, respectively, as compared to the medical datasets where the class imbalance levels are of the order of 20:80, approximately. Hence, there is a need to investigate the performance of region-based losses for thin RoIs and extreme class imbalance in PL detection.

### 2.3. Compound Loss Functions

Compound loss functions combine the distribution and overlap-based loss functions to tackle the class imbalance in semantic segmentation problems. These loss functions also introduce additional weighting parameters to weight the constituent losses and thus, require tuning to achieve optimal performance. Furthermore, these losses have also been applied to medical segmentation tasks only [22,33] where the class imbalance levels are less severe and RoIs are more small and compact than the PL detection tasks. Apart from the tuning of weights, the learning rates (LRs) are also difficult to configure and optimize for the compound losses due to the varying nature of the constituent losses. Empirically, region-based losses require lower LRs in comparison to the distribution-based losses. 

As far as PL detection is concerned, the work in [8] utilizes a compound loss based on BCE loss and the Jaccard loss [34] with a weighting parameter λ to weight the Jaccard loss. The study, however, lacks a thorough investigation of the proposed compound loss with other losses.

In summary, PL detection suffers from a severe class imbalance (2:98, approximately) problem due to thin RoIs, which adversely affects the detection performance. The current literature on PL detection largely utilizes the distribution-based BCE loss and its variants for tackling the class imbalance issue without an investigation of alternate loss functions. Empirical investigation of the popular BBCE loss (Section 4.3.2) for PL detection task reveals that it fails to achieve an optimal trade-off for the evaluation parameters. Region-based losses have demonstrated better performances than distribution-based losses for the medical segmentation tasks with small RoIs and class imbalance levels of (20:80), approximately. However, these losses have not been investigated for thin RoIs and more severe class imbalance levels. Moreover, these region-based losses require a careful adjustment of additional weighting hyperparameters. Hence, there is a need for an appropriate loss function which can address the severe class imbalance and thin RoIs for PL detection while achieving an optimal trade-off for the characteristic evaluation parameters along with minimal optimization effort. 

## 3. Method

### 3.1. Focal Phi Loss

The Matthews correlation coefficient (MCC) or the Phi coefficient is widely used in machine learning and bioinformatics for assessing the quality of binary classifications. It measures the degree of correlation between the actual and the predicted values and returns a value in the range of (−1, 1), where −1 and 1 indicate a complete disjoint and a perfect prediction, respectively [35]. The Phi coefficient is defined by Equation (7).

MCC = ((TP × TN) − (FP × FN))/√ ((TP + FP) (TP + FN) (TN + FP) (TN + FN))(7)

where TP, TN, FP and FN are the true positive, true negative, false positive and false negative entries of the confusion matrix, respectively. It can be seen from Equation (7) that MCC considers all the entries of the confusion matrix in its calculation as opposed to the Dice coefficient and the Tversky index, which do not consider the true negative values (TN). The MCC loss [26] can be defined in Equation (8) as:

L_MCC_ = (1 − MCC) (8)

Similar to DL and TL, MCC loss has also been applied to medical image segmentation with small RoIs and less severe class imbalance [26]. An experimental investigation (Section 4.3.1) of the MCC loss for our PL detection task, with thin RoIs and more severe class imbalance, reveals that MCC loss outperforms the baseline BBCE loss in dice scores (DSC) and precision values but results in low sensitivity (recall) and accuracy values. This suggests that there is a need to further improve the classification of the hard thin RoI samples against the easy background samples. Hence, to classify the thin RoIs more accurately while achieving an optimal trade-off for other evaluation parameters, we modulate the linear nature of L_MCC_ by introducing an exponent γ. In order to differentiate our proposed loss function from L_MCC_, we name it the focal Phi loss (FPL) instead of the focal Matthews correlation coefficient loss. We define our Focal Phi Loss (FPL) as:

L_FPL_ = (1 − MCC) ^γ^ (9)

where γ lies in the range of (0, 3] and

MCC = ((TP × TN) − (FP × FN) + ε)/√ ((TP + FP) (TP + FN) (TN + FP) (TN + FN) + ε)(10)

It should be noted that unlike L_MCC_, we incorporate numerical stability to the MCC metric calculation in Equation (10) by introducing a smoothing factor ε to prevent undefined values. The focal parameter γ in our FPL (Equation (9)) allows the loss to focus more on the hard classes that are detected with lower probability. The idea of applying a focal exponent γ to the loss function is inspired from the works [21,30], which facilitates better control between easy and hard training examples.

As far as skewness is concerned, our L_FPL_ handles it automatically by assigning the class sample size ratios of √B/A and √A/B to TN and TP, respectively, during MCC calculation, where A and B specify the majority and minority classes, respectively [36]. That is if A = TN + FP and B = TP + FN then FP = A − TN and FN = B − TP, so, Equation (10) becomes:

MCC = (−√AB + √B/A TN + √A/B TP + ε)/√ ((TP + A − TN) (TN + B − TP) + ε)(11)

Hence, there is no need to assign additional weighting parameters for addressing the class imbalance issue as is the case in distribution-based losses (Equations (2)–(4)).

### 3.2. Network Architecture

**Figure 1:**
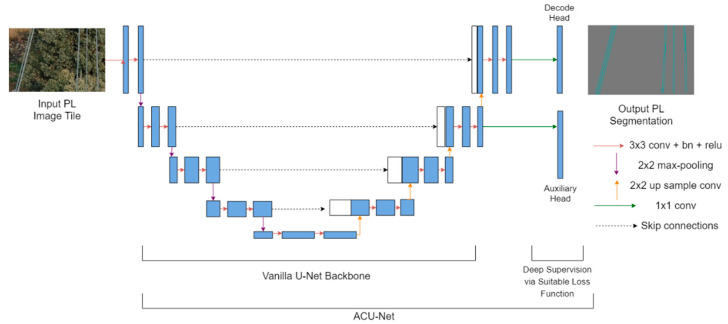
Proposed auxiliary classifier-U (ACU)-Net architecture with additional auxiliary head for better feature learning and model convergence.

To achieve an optimal trade-off for the characteristic evaluation parameters and to better optimize the learning process, we propose an auxiliary classifier U-Net (ACU-Net) as the baseline segmentation network and optimize it using our proposed FPL (and other suitable loss functions for experiments in Section 4.3.2) via error backpropagation. Our proposed architecture is based on the popular vanilla U-Net [25], which is an encoder–decoder-based deep segmentation network with characteristic skip connections. The encoder comprises of a down sampling path to extract representative features from the input image using 3 × 3 convolutions and 2 × 2 max pooling operations. On the other hand, the decoder up samples the features extracted by the encoder with 2 × 2 up sampling convolutions and 3 × 3 convolutions so that the features comprise of meaningful contextual information for effective pixel classification. Skip connections between various stages of the encoder and decoder facilitate the fusion of high-resolution encoder features with low resolution decoder features for more semantically complete outputs. Our ACU-Net (Figure 1) improves the vanilla U-Net [25] by incorporating an additional 1 × 1 convolutional head to the output of the second last decoding stage in the vanilla U-Net. This additional convolutional head, termed as the auxiliary classifier head, is used to supervise the backbone feature learning task with a suitable loss function (our proposed FPL or other loss functions as discussed in Section 4.3.2). The main task of pixel level PL detection is supervised by the 1 × 1 convolutional decode head, at the end of the last decoding stage, similar to the vanilla U-Net. Same loss functions are employed for both the heads but are effectively weighted. The sigmoid activation is applied to the outputs of 1 × 1 decode and auxiliary heads inside the loss function. Hence, the vanilla U-Net acts as the feature learning backbone network in our architecture. The addition of an auxiliary classifier and loss weighting for multiple losses is a deep supervision trick [37,38] that helps to optimize the feature learning process and improve the model performance. The auxiliary classifier head is only utilized during training and dropped during the inference phase.

## 4. Experiments

### 4.1. Dataset

We have utilized two benchmark PL datasets in this study, namely the Mendeley PL Dataset [39] and the Power Line Dataset of Urban Scenes (PLDU) [40]. These datasets contain the visual light images of electrical power lines captured by a UAV along with their corresponding pixel-wise ground truth (GT) annotations. The Mendeley PL dataset is composed of 400 images of size 512 × 512, out of which 200 images contain the PLs while the remaining 200 images only contain the background class (Bg) with no PLs in them. We split this dataset according to the 80:20 train-validation split so that the training dataset contains 320 images while the validation dataset contains the remaining 80 images. We employ the stratified sampling method to split the dataset into train-validation sets, following which, both the training and the validation sets contain half of the images which include the PLs while the remaining half are the pure background images. The class imbalance level (PL:Bg), denoted by rho *ρ*, for this dataset is 0.073:99.92. The PLDU dataset is already split into 453 training and 120 validation images. The images in this dataset are either of size 560 × 360 or of size 360 × 560. The class imbalance in the PLDU dataset is 1.18:98.82. Thus, the class imbalance in the Mendeley PL dataset is more severe than the PLDU dataset. The class imbalance levels are calculated on the entire dataset (training + validation) according to the formula [11] defined as:

*ρ* = max_i_(|Ci|)/min_i_(|Ci|) (12)

where Ci is a set of examples in class *i*, and max_i_(|Ci|) and min_i_(|Ci|) return the maximum and minimum class size over all *i* classes, respectively. The details of the PL benchmark datasets discussed are summarized in Table 1.

**Figure 2:**
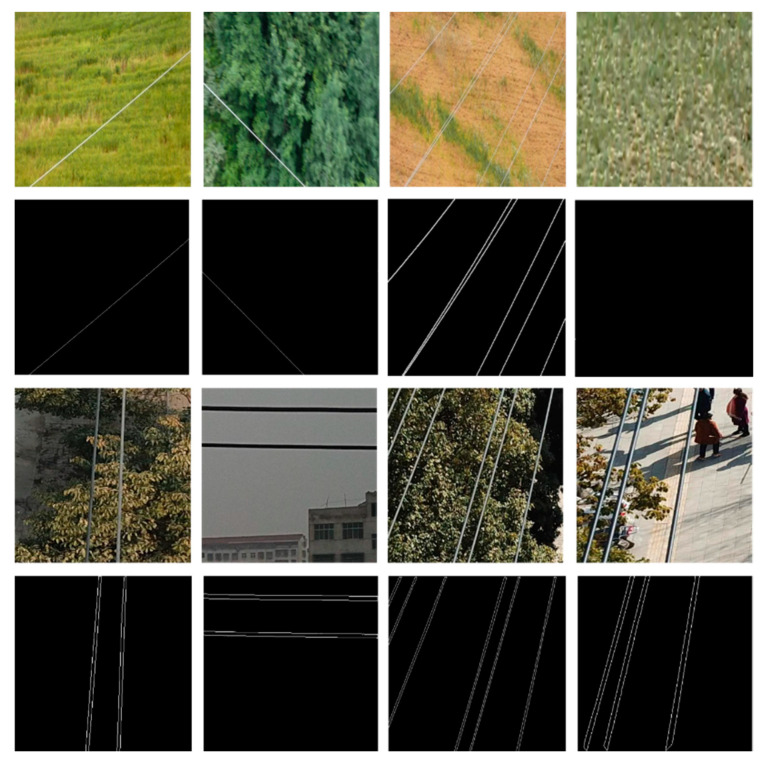
Example images of power lines and their corresponding ground truth annotations from the PL benchmark datasets. Top two rows: Mendeley PL dataset [39]; bottom two rows: PLDU [40].

Some of the images from these datasets and their corresponding ground truth annotations are illustrated in Figure 2. It should be noted that the thin RoIs in PLDU (Figure 2 last row) are thinner as compared to those of the Mendeley PL dataset (Figure 2 second row). This is because the thin RoIs in PLDU are specified by precise boundaries while the area between those boundaries do not contain any PL pixels. Hence, each PL in PLDU is actually represented by two thin boundary lines. On the other hand, the PLs in the Mendeley PL dataset are specified by a single line of pixels signifying the thin RoI. Thus, it is more difficult to segment and localize the PLs in PLDU accurately as compared to those in the Mendeley PL dataset even though the class imbalance in the Mendeley PL dataset is higher than PLDU.

The PL detection task is framed as a binary semantic segmentation problem with the aim of accurately classifying and localizing the minority class PL pixels against the majority class background pixels. Since the size of the datasets is rather small for training a deep segmentation network, the following on the fly data augmentations are applied to the images in both the training datasets.

- Scaling the images for a ratio range of (0.5, 1.5);
- Random rotation in the range of (45.0, 315.0) with a probability of 50%;
- Horizontal flipping with a probability of 50%;
- Resizing the images to 256 × 256;
- Random brightness with a delta of 32;
- Random contrast in the range of (0.5, 1.5);
- Random saturation in the range of (0.5, 1.5);
- Random hue with a delta of 0.5;
- Image padding for the resized images with size 256 × 256.

As far as the validation datasets are concerned, only the scaling and flipping data transforms are applied during the validation phase. We utilize the *mmsegmentation* framework [41], a Pytorch-based framework, to implement our proposed FPL function along with the ACU-Net for PL segmentation. The GT mask images are transformed into the mmsegmentation format with the following class to color palette mapping:

- PL class pixels with a color of (6, 230, 230);
- Bg class pixels with a color of (120, 120, 120).

The network parameters are initialized with zero mean and unit standard deviation Gaussian distribution. The proposed ACU-Net (Figure 1) is trained with various loss functions discussed in Section 2, on the two PL datasets (Table 1) for 20K iterations and a batch size of two images. The same loss functions are employed for the decode and auxiliary classifier heads, but the loss weights are set to 1.0 and 0.4, respectively. The Adam optimizer is employed for model training with an initial LR of 1 × 10^−3^ and a weight decay of 0.0001 for all the loss functions with the exception of region-based losses (DL, TL and FTL functions). For region-based losses, lower LRs are required for model convergence, that is, 5 × 10^−5^ for the Mendeley PL dataset and 1 × 10^−7^ for PLDU. The LR of the decode and auxiliary heads is set to 10 times to that of the backbone to achieve faster convergence. Validation and model checkpointing is performed after every 200 iterations during the model training. These parameters are identified via a grid search method. No transfer learning is applied for training ACU-Net in order to present a fair evaluation of the proposed ACU-Net and the proposed FPL function against the vanilla U-Net and other loss functions, respectively. All the experiments are performed using Tesla K80 GPU compute from Google Colab.

### 4.2. Evaluation Parameters

To the best of authors’ knowledge, almost all the studies regarding PL detection use the dice scores (DSC) (also known as the F1-score), precision, true positive rate (TPR) (also known as recall or sensitivity), false discovery rate (FDR) and accuracy [4,6,7,8,12,13,14]. These evaluation parameters are defined as:

DSC or F1-score = 2TP/(2TP + FP +FN)(13)

Precision = TP/(TP + FP)(14)

TPR or Recall or Sensitivity = TP/(TP + FN)(15)

FDR = FP/(FP + TP)(16)

Accuracy = (TP + TN)/(TP + TN + FP + FN)(17)

where TP, TN, FP and FN represent the true positive, true negative, false positive and false negative entries of the confusion matrix, respectively. TPR and FDR specify the percentage of successful and failure detections of the foreground PL class, respectively while DSC and accuracy are comprehensive indicators of the overall model performance in general. It is worth noting that none of these evaluation measures assess the performance of the trained model for the major Bg class separately. Hence, to assess the performance of the major Bg class in comparison to the minor PL class we suggest monitoring two extra parameters, namely the true negative rate (TNR) (also known as specificity) [20] and the DSC for the Bg class defined as:

TNR or Specificity = TN/(TN + FP)(18)

DSC_Bg_ = 2TN/(2TN + FP + FN) (19)

We denote the class-wise DSC by DSC_PL_ (Equation (13)) and DSC_Bg_ (Equation (19)) to distinguish the foreground and background PL and Bg classes, respectively. After the training of all the algorithms, the results of PL detection are compared to the GT pixel annotations in the light of above evaluation parameters for performance evaluation.

### 4.3. Experimental Results

We divide the results section into four different parts, that is, (1) proposed FPL function results with different values of its constituent parameter γ using the proposed ACU-Net, (2) investigation of various state-of-the-art loss functions for class imbalance to the PL detection task with the proposed ACU-Net and its comparative analysis with our proposed FPL, (3) ablation study results of the proposed ACU-Net, and, (4) analysis and deductions of the obtained results. The best values for each evaluation parameter are written in bold in the tabular results. The code implementation of all the conducted experiments is available at the link mentioned in the Supplementary Materials. 

#### 4.3.1. Focal Phi Loss (FPL) Results

The results of training the ACU-Net with the proposed FPL for the segmentation of PLs in the Mendeley PL dataset and PLDU are summarized in Table 2. We experiment with various values of the focal parameter γ to understand its effect on the proposed FPL function. γ is increased in uniform steps of 0.5 in the range of (0, 3]. We also experiment with γ = 0.75 owing to its popular choice in the FTL function [21]. It should be noted that when γ = 1.0, our proposed FPL is equivalent to the MCC loss [26].

**Figure 3:**
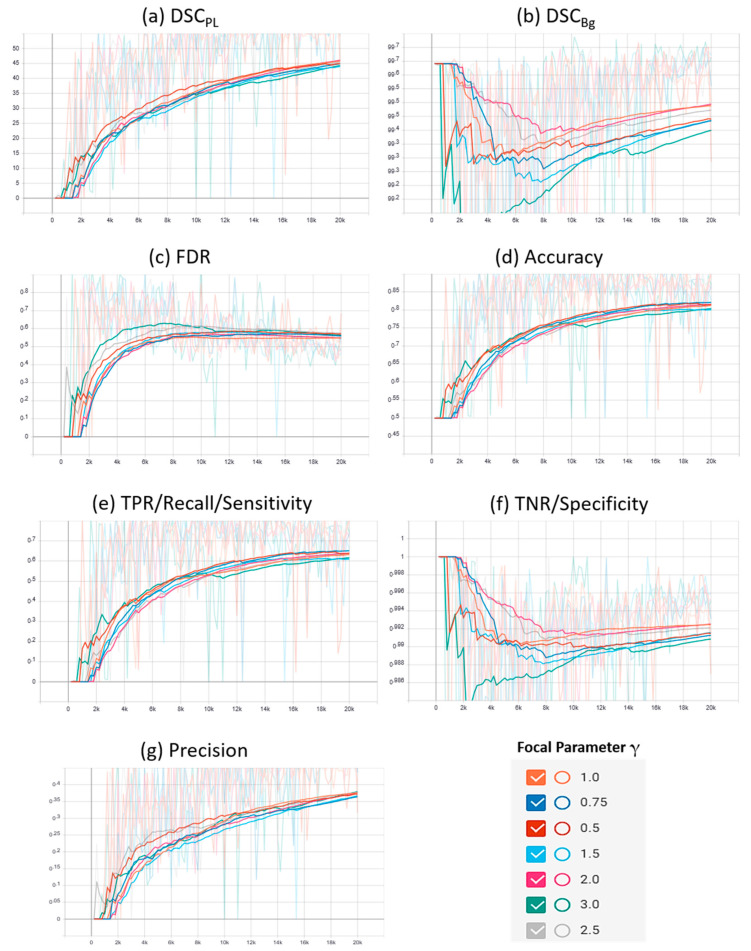
Characteristic evaluation parameters for the proposed focal Phi loss (FPL) function (with various values of focal parameter γ) and the Matthews correlation coefficient (MCC) loss (with γ = 1.0) on the Mendeley PL dataset.

**Figure 4:**
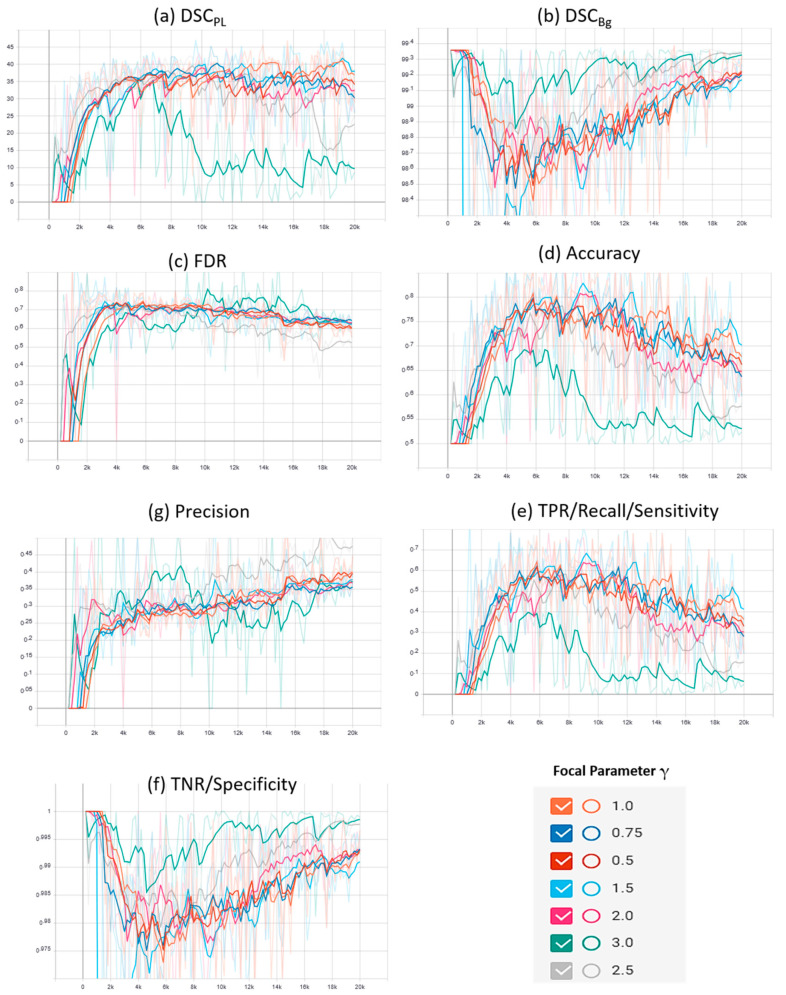
Characteristic evaluation parameters for the proposed focal Phi loss (FPL) function (with various values of focal parameter γ) and the MCC loss (with γ = 1.0) on PLDU.

A plot of the characteristic evaluation parameters against the training iterations (20K) with model validation at every 200 iterations is presented graphically in Figure 3 and Figure 4 for Mendeley PL dataset and PLDU, respectively. Exponential moving average with smoothing parameter S_α_ is applied to smooth the graphs and highlight the trend in the data points. S_α_ is set to 0.6 and 0.9 for PLDU and Mendeley PL datasets, respectively.

#### 4.3.2. Investigation of Various State-of-the-Art Loss Functions for PL Detection

In this section, we examine five other state-of-the-art loss functions, commonly used for class imbalance problems, on the PL benchmark datasets and compare the results with our proposed FPL function. The five different loss functions evaluated in this study are: BBCE loss, MCC loss, DL, TL and FTL. The parameters for these loss functions are obtained after a grid search. To the best of our knowledge, no research work has investigated the application of different loss functions for the highly imbalanced PL detection task with thin RoIs. All the studies on PL detection mainly rely on BCE loss [6,14] and its variants [4,7,12] to handle the class imbalance problem, with the BBCE loss being the baseline in the majority of these works. The same ACU-Net network architecture is trained with each of these loss functions and the characteristic evaluation parameters are monitored. The results for the Mendeley PL dataset and PLDU are summarized in Table 3 and Table 4, respectively. 

**Figure 5:**
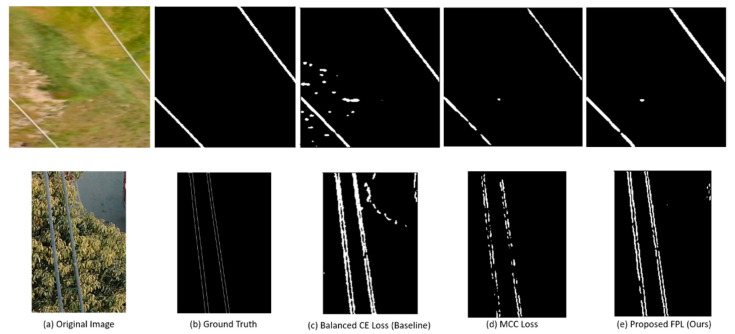
Power line detection results on a sample image from top row: the Mendeley PL dataset, bottom row: PLDU. From (**a**–**e**): original, ground truth, balanced cross entropy (CE) loss (baseline), MCC loss, proposed FPL (ours).

A qualitative assessment of our proposed FPL in comparison to the baseline BBCE loss and the original MCC loss is depicted via the results in Figure 5. Figure 5 presents a sample image from each PL dataset and its GT annotation, as well as the results of three top performing methods according to Table 3 and Table 4, that is the baseline BBCE, the original MCC loss and our proposed FPL.

We can observe that for both the sample images, the (a) baseline BBCE loss function results in a high number of FP PL predictions which do not correspond to the actual PL pixels in the GT annotations. The (b) MCC loss addresses these FPs effectively but has a lower recall rate and therefore detects PLs with disconnected pixels. The superiority of our (c) proposed FPL function to other methods can clearly be depicted in these results as the PL predictions are strongly connected and comprise of little to no FPs. Hence, the PLs detected with our proposed FPL are closest to the (b) GT annotations.

**Figure 6:**
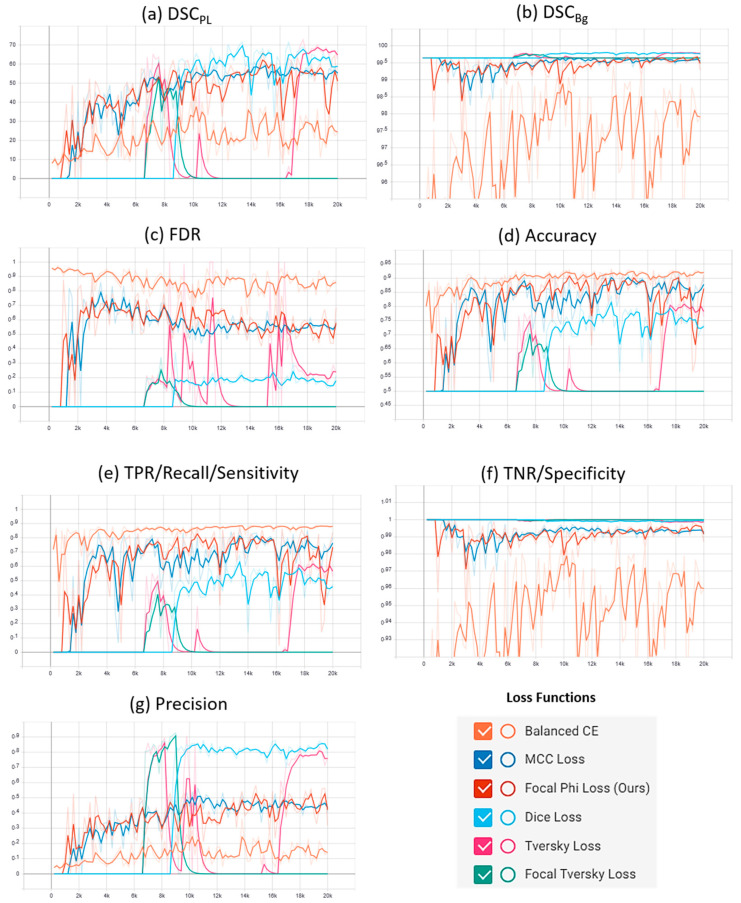
Comparative analysis of the proposed focal Phi loss (FPL) function with various loss functions for class imbalance on the Mendeley PL dataset.

**Figure 7:**
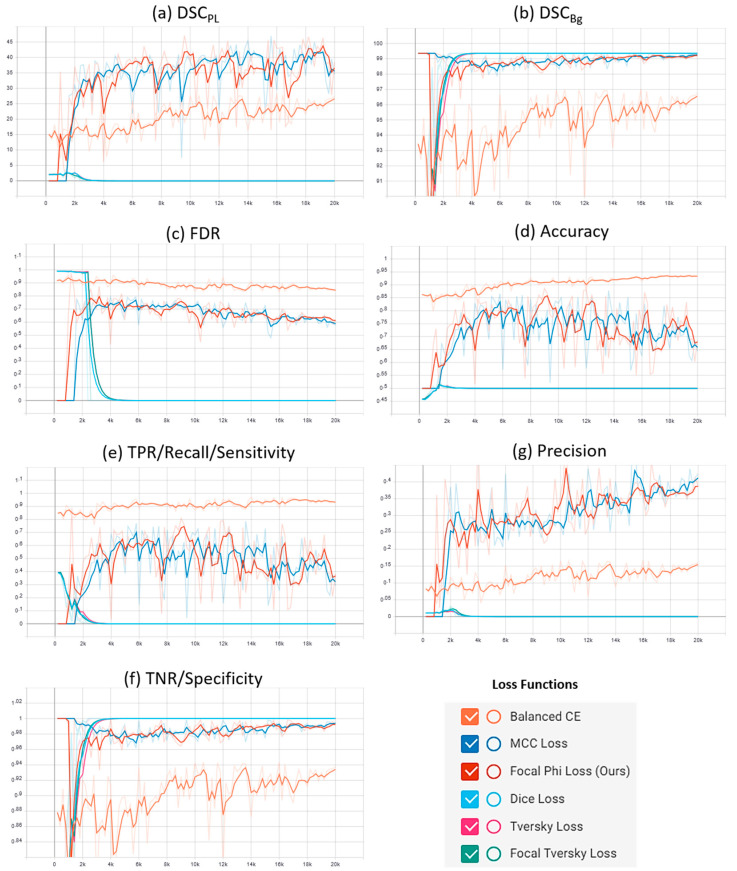
Comparative analysis of the proposed focal Phi loss (FPL) function with various loss functions for class imbalance on PLDU.

Supplementary plots of the characteristic evaluation parameters against the training iterations (20K) for the mentioned loss functions are presented graphically in Figure 6 and Figure 7 for the Mendeley PL dataset and PLDU, respectively. Exponential smoothing with S_α_ = 0.6 is applied to the plot results for both the datasets.

#### 4.3.3. Ablation Study

The novel contributions of this study can be divided into two parts: (a) focal Phi loss (FPL) function for class imbalanced PL detection and thin RoIs segmentation; and (b) U-Net with auxiliary classifier (ACU-Net) for better feature learning and faster model convergence. The proposed FPL function results have already been depicted in Section 4.3.1 and Section 4.3.2. In this section, we analyze the effects of the ACU-Net for the PL detection task by conducting an ablation study and documenting the results for the Mendeley PL dataset and PLDU in Table 5 and Table 6, respectively.

#### 4.3.4. Analysis and Discussion

##### Comparison of FPL with MCC Loss

The results in Table 2 depict that an optimal trade-off for the characteristic evaluation parameters is achieved for the Mendeley PL dataset and PLDU when the focal parameter γ is equal to 0.5 and 1.5, respectively. The results achieved with our proposed FPL (with γ = 0.5) on the Mendeley PL dataset are better than those achieved with MCC loss (γ = 1.0) due to highest class-wise dice scores (~4% and 0.10% higher DSC_PL_ and DSC_Bg_, respectively), highest precision (~7%), highest TNR values (~0.2%) and lowest FDR values (~7% lower) with a minor degradation of ~1% in the accuracy and TPR values. For PLDU, our proposed FPL (with γ = 1.5) outperforms the MCC loss (γ = 1.0) with the highest DSC_PL_ (~1% higher), highest TPR (~5%) and highest accuracy values (~3%), while our FPL (with γ = 3.0) outperforms MCC loss with the highest DSC_Bg_ (~1% higher), highest TNR (~1%), highest precision (~5%) and lowest FDR values (~5% lower). For PLDU, γ = 1.5 is chosen as the optimal value for FPL as it yields the highest results for the PL class parameters as opposed to γ = 3.0 which yields the best results for the Bg class parameters. Table 2 also reveals that for both the datasets, the results obtained for the characteristic evaluation parameters pertaining to the PL class (DSC_PL_ and TPR) and overall model performance (accuracy, precision and FDR) are better when γ < 1 while the results of the characteristic evaluation parameters for the Bg class (DSC_Bg_ and TNR) are better with γ > 1. It is also noteworthy that the overall results of all the evaluation parameters are higher for the Mendeley PL dataset as compared to PLDU. This is because the thin RoIs in PLDU are harder to segment (Section 4.1.) as compared to the thin RoIs in the Mendeley PL dataset.

Similar deductions can be made by visualizing the plots of the characteristic evaluation parameters in Figure 3 and Figure 4 that our proposed FPL, with γ = 0.5 (Mendeley PL dataset) and γ = 1.5 (PLDU), outperforms the MCC loss (γ = 1.0) by achieving superior values (high peaks) for (a) DSC_PL_ and (b) TPR for the thin PL class while maintaining acceptable values for the Bg class, with metrics (b) DSC_Bg_ and (f) TNR, for both the datasets. Apart from these per class results, our proposed FPL also yields an overall better model performance for both the datasets with higher (c) accuracy, higher (g) precision and lower (c) FDR values as compared to the MCC loss (γ = 1.0).

##### Comparative Analysis of FPL with State-of-the-Art Loss Functions

Table 3 reveals that our proposed FPL function outperforms the baseline BBCE loss on the Mendeley PL dataset by ~16% for DSC_PL_, ~0.4% for DSC_Bg_, ~1% for TNR and ~19% for precision and FDR values with a minor degradation in the TPR (~3%) and accuracy (~1%) values. For PLDU in Table 4, our proposed FPL outperforms the BBCE loss by ~16% on DSC_PL_, ~2% on DSC_Bg_, 4% on TNR and ~15% for precision and FDR values. Although, our proposed FPL lags behind the BBCE loss in terms of accuracy (~7%) and especially TPR values (~18%) for PLDU, it still achieves a better trade-off amongst all the evaluation parameters as compared to the BBCE loss. Furthermore, the region-based DL and TL achieve the highest DSC_PL_ (~70%) and DSC_Bg_ (~99%) values with lower FDR (~20%) and the best precision–recall trade-off for the Mendeley PL dataset in Table 3, but these losses lag behind our proposed FPL in terms of TPR and accuracy values by ~11% and ~7%, respectively. The FTL yields the best FDR and precision values but falls back on other evaluation parameters for the Mendeley PL dataset. As far as PLDU is concerned, the region-based DL, TL and FTL yield extremely poor values for the PL class (DSC_PL_, TPR) and the overall model performance (accuracy, precision and FDR) while being biased towards the major Bg class with higher DSC_Bg_ and TNR values. The comparison of the proposed FPL with the MCC loss has already been discussed in first part of Section 4.3.4. 

The TL and FTL region-based losses, as well as the BBCE loss, require the tuning of multiple hyperparameters (α, β for TL, α, β and γ for FTL and class weights β and 1 − β for BBCE loss) for model training. Although DL does not require the tuning of additional hyperparameters (α and β are fixed to 0.5), it tends to ignore the TNs in its calculation and thus results in low specificity or TNR values. As a result, our proposed FPL is more promising for class imbalance and thin RoIs segmentation, as it addresses these issues effectively. Similar to the FPL results in Table 2, the overall performance of other loss functions is higher for the Mendeley PL dataset in comparison to PLDU due to the thinner RoIs in PLDU.

Similar deductions can be made by visualizing the plots in Figure 6 and Figure 7 that our proposed FPL provides a better trade-off for the evaluation parameters in comparison to other loss functions on both the PL datasets. Another interesting trend to be noted here is that in BBCE loss as the accuracy increases, the corresponding DSC_PL_ values decrease for both the datasets. We deduce that this is due to a higher number of FPs as FDR values for the BBCE loss are also higher than for other losses. Thus, there is an inverse relationship between the FDR and DSC_PL_ values and an optimal model must therefore have a lower FDR in order to perform efficiently on the minority PL class.

##### Comparative Analysis of Vanilla U-Net with ACU-Net

The results in Table 5 and Table 6 reveal that our proposed ACU-Net, which simply adds an auxiliary classifier head to the vanilla U-Net, outperforms the vanilla U-Net for the majority of the evaluation parameters in all the loss function experiments, on both the PL datasets, with the only exception of BBCE loss on the Mendeley PL dataset. The BBCE loss with the Mendeley PL dataset is the only case where the vanilla U-Net performs better than our proposed ACU-Net (Table 5). Apart from this exception, a general improvement can be seen for the majority of the evaluation parameters in Table 5, when ACU-Net is employed for PL detection instead of vanilla U-Net. The improvement is approximately in the range of 1–10% for all the losses on both the PL datasets.

## 5. Conclusions and Future Work

In this study, we examined the PL detection task, with thin RoIs, from the class imbalance perspective. We proposed a novel focal Phi loss (FPL) function for the thin RoIs segmentation of PL pixels against the background in two highly imbalanced PL datasets, i.e., the Mendeley PL dataset and the Power Line Dataset of Urban Scenes (PLDU). The proposed FPL introduced a focal component to the MCC loss for achieving an optimal trade-off for the characteristic evaluation parameters. We also provided an in-depth comparative analysis of our proposed FPL against the established loss function baselines with a standardized deep network architecture. Our experiments demonstrate the significance of the choice of loss function when dealing with class imbalance in the PL detection task. Moreover, we also improved the vanilla U-Net by incorporating an additional auxiliary classifier head (ACU-Net) for learning the features of thin PLs effectively and for faster model convergence. Our proposed FPL and ACU-Net outperformed the baseline BBCE loss function for class imbalance in PL detection and the vanilla U-Net architecture, respectively, by achieving an optimal trade-off for the characteristic evaluation parameters, with particularly higher values for the foreground PL class dice scores (DSC_PL_), precision and FDR values. Compared to other loss functions, our proposed FPL is simple and effective as it requires tuning for only a single focal parameter (γ). Since FPL yields remarkable results for thin RoIs segmentation with class imbalance, its application can be extended to various research domains where thin RoIs segmentation is of the utmost importance. These domains include but are not limited to mooring lines monitoring and maintenance in the marine vessels, road-lane line detection in autonomous vehicles, sperm and bacterial flagella detection in medicine, automated cracks detection to ensure architectural sustainability, etc. Furthermore, FPL can also pave the way for the design of a generic thin RoIs detector which can save the cost of acquiring a separate application-specific detector for various application areas.

Currently, the proposed FPL lags behind the baseline BBCE loss in accuracy values. We believe that this problem can be alleviated, and the accuracy of FPL can be improved by combining it with the BCE loss as BCE loss is designed to maximize the accuracy values. Apart from the proposed improvement of combining the FPL with BCE loss, an investigation of the application of FPL for application-specific deep PL detectors, instead of a standardized generic U-Net architecture, is also to be carried out as part of potential future work. We also anticipate investigating the choice of alternate auxiliary classifier head architectures for the proposed ACU-Net and observe their performances for different application domains.

## Figures and Tables

**Table 1:** Detailed information of the power line (PL) detection datasets used in this study.

S.#	Dataset	Train	Val	Image Size	Annotation Type	Class Imbalance Level (*ρ*)
1	Mendeley PL Dataset [39]	320	80	512 × 512	Pixel	0.073:99.92
2	Power line Dataset of Urban Scenes (PLDU) [40]	453	120	560 × 360 or 360 × 560	Pixel	1.18:98.82

**Table 2:** Experimental results of the proposed focal Phi loss (FPL) on the Mendeley PL and PLDU validation datasets with ACU-Net. DSC: dice score; TPR: true positive rate; TNR: true negative rate; FDR: false detection rate.

SN	Dataset	Focal Parameter	DSC_PL_	DSC_Bg_	TPR	TNR	FDR	Precision	Accuracy
1	Mendeley PL Dataset	γ = 0.5	**63.025**	**99.65**	81.33	**99.435**	**47.965**	**52.035**	90.385
		γ = 0.75	58.38	99.57	81.775	99.28	54.275	45.725	90.525
		γ = 1.0	58.145	99.56	**82.97**	99.25	54.855	45.145	**91.11**
		γ = 1.5	59.55	99.595	81.615	99.32	52.67	47.33	90.47
		γ = 2.0	60.045	99.61	79.45	99.37	51.235	48.765	89.41
		γ = 2.5	60.865	99.63	78.78	99.41	49.8	50.2	89.1
		γ = 3.0	60.6	99.61	80.88	99.36	50.835	49.165	90.12
2	Power line Dataset of Urban Scenes (PLDU)	γ = 0.5	41.35	98.735	65.72	97.935	68.08	31.92	81.83
		γ = 0.75	41.635	98.675	70.325	97.76	69.765	30.235	84.045
		γ = 1.0	42.46	98.725	68.905	97.88	68.03	31.97	83.39
		γ = 1.5	**43.065**	98.705	**73.615**	97.78	69.165	30.835	**85.695**
		γ = 2.0	38.52	98.455	68.435	97.36	71.785	28.21	82.9
		γ = 2.5	38.215	98.55	64.035	97.595	70.595	29.40	80.815
		γ = 3.0	41.47	**99.06**	50.97	**98.76**	**62.075**	**37.925**	74.865

**Table 3:** Experimental results comparison of various loss functions on Mendeley PL validation dataset with 80 images.

SN	Loss Function	Parameters	Learning Rate	DSC_PL_	DSC_Bg_	TPR	TNR	FDR	Precision	Accuracy
1	Balanced Cross Entropy (BBCE) Loss (baseline) [29]	β = 0.98 (PL class), 1 − β = 0.02 (Bg)	1 × 10^−3^	46.785	99.23	**84.605**	98.67	67.48	32.52	**91.655**
2	Matthews Correlation Coefficient (MCC) Loss [26]	γ = 1.0	1 × 10^−3^	58.145	99.56	82.97	99.25	54.855	45.145	91.11
3	Focal Phi Loss (FPL) (Ours)	γ = 0.5	1 × 10^−3^	63.025	99.65	81.33	99.435	47.965	52.035	90.385
4	Tversky Loss (TL) [20]	α = 0.3, β = 0.7	5 × 10^−5^	**72.78**	**99.81**	70.60	99.83	24.90	75.10	85.22
5	Dice Loss (DL) [23]	α = 0.5, β = 0.5	5 × 10^−5^	71.37	**99.81**	66.325	99.85	22.69	77.31	83.09
6	Focal Tversky Loss (FTL) [21]	α = 0.3, β = 0.7, γ = 0.75	5 × 10^−5^	52.67	99.755	37.94	**99.955**	**13.875**	**86.125**	68.945

**Table 4:** Experimental results comparison of various loss functions on PLDU validation dataset with 120 images.

SN	Loss Function	Parameters	Learning Rate	DSC_PL_	DSC_Bg_	TPR	TNR	FDR	Precision	Accuracy
1	Balanced Cross Entropy (BBCE) Loss (baseline) [29]	β = 0.98 (PL class), 1 − β = 0.02 (Bg)	1 × 10^−3^	27.145	96.715	**91.485**	93.745	84.05	15.95	**92.615**
2	Matthews Correlation Coefficient (MCC) Loss [26]	γ = 1.0	1 × 10^−3^	42.46	**98.725**	68.905	**97.88**	**68.03**	**31.97**	83.39
3	Focal Phi Loss (FPL) (Ours)	γ = 1.5	1 × 10^−3^	**43.065**	98.705	73.615	97.78	69.165	30.835	85.695
4	Tversky Loss (TL) [20]	α = 0.3, β = 0.7	1 × 10^−7^	3.52	88.47	29.42	80.05	98.13	1.87	54.74
5	Dice Loss (DL) [23]	α = 0.5, β = 0.5	1 × 10^−7^	3.89	93.23	20.06	88.22	97.85	2.15	54.14
6	Focal Tversky Loss (FTL) [21]	α = 0.3, β = 0.7, γ = 0.75	1 × 10^−7^	3.62	89.31	28.35	81.43	98.07	1.93	54.89

**Table 5:** Experimental results comparison of the proposed ACU-Net and vanilla U-Net model with various loss functions on Mendeley PL validation dataset with 80 images.

SN	Loss Function	Parameters	Learning Rate	Model	DSC_PL_	DSC_Bg_	TPR	TNR	FDR	Precision	Accuracy
1	Balanced Cross Entropy (BBCE) Loss (baseline) [29]	β = 0.98 (PL class), 1 − β = 0.02 (Bg)	1 × 10^−3^	ACU-Net (Ours)	46.785	99.23	84.60	98.67	67.48	32.52	91.655
				Vanilla U-Net	**50.555**	**99.38**	**86.38**	**98.86**	**64.1**	**35.9**	**92.62**
2	Matthews Correlation Coefficient (MCC) Loss [26]	γ = 1.0	1 × 10^−3^	ACU-Net (Ours)	**58.145**	**99.56**	**82.97**	99.25	54.85	45.145	**91.11**
				Vanilla U-Net	56.975	99.585	73.87	**99.36**	**51.67**	**48.33**	86.62
3	Focal Phi Loss (FPL) (Ours)	γ = 0.5	1 × 10^−3^	ACU-Net (Ours)	**63.025**	**99.65**	81.33	**99.43**	**47.965**	**52.035**	**90.385**
				Vanilla U-Net	56.240	99.525	**83.320**	99.170	57.250	42.750	91.245
4	Tversky Loss (TL) [20]	α = 0.3, β = 0.7	5 × 10^−5^	ACU-Net (Ours)	**72.78**	**99.81**	**70.60**	99.83	24.90	75.10	**85.22**
				Vanilla U-Net	71.815	99.805	68.510	**99.840**	**24.555**	**75.445**	84.175
5	Dice Loss (DL) [23]	α = 0.5, β = 0.5	5 × 10^−5^	ACU-Net (Ours)	**71.37**	**99.81**	**66.325**	99.85	**22.69**	**77.31**	**83.09**
				Vanilla U-Net	70.720	99.805	65.31	**99.860**	22.890	77.110	82.590
6	Focal Tversky Loss (FTL) [21]	α = 0.3, β = 0.7, γ = 0.75	5 × 10^−5^	ACU-Net (Ours)	**52.67**	**99.755**	**37.94**	99.955	13.875	86.125	**68.945**
				Vanilla U-Net	40.110	99.720	26.070	**99.975**	**13.060**	**86.940**	63.020

**Table 6:** Experimental results comparison of the proposed ACU-Net and vanilla U-Net model with various loss functions on PLDU with 120 images.

SN	Loss Function	Parameters	Learning Rate	Model	DSC_PL_	DSC_Bg_	TPR	TNR	FDR	Precision	Accuracy
1	Balanced Cross Entropy (BBCE) (baseline) [29]	β = 0.98 (PL class), 1 − β = 0.02 (Bg)	1 × 10^−3^	ACU-Net (Ours)	**27.145**	**96.715**	**91.485**	**93.745**	**84.05**	**15.95**	**92.615**
				Vanilla U-Net	23.245	96.315	82.41	93.115	86.400	13.60	87.76
2	Matthews Correlation Coefficient (MCC) Loss [26]	γ = 1.0	1 × 10^−3^	ACU-Net (Ours)	**42.46**	**98.725**	68.905	**97.88**	**68.03**	**31.97**	83.39
				Vanilla U-Net	38.340	98.410	**72.905**	97.215	73.555	26.445	**85.055**
**3**	Focal Phi Loss (FPL) (Ours)	γ = 1.5	1 × 10^−3^	ACU-Net (Ours)	**43.065**	98.705	**73.615**	97.78	**69.165**	**30.835**	**85.695**
				Vanilla U-Net	41.625	**98.760**	66.720	**97.980**	69.280	30.720	82.345
4	Tversky Loss (TL) [20]	α = 0.3, β = 0.7	1 × 10^−7^	ACU-Net (Ours)	**3.52**	88.47	**29.42**	80.05	**98.13**	**1.87**	**54.74**
				Vanilla U-Net	2.905	**90.865**	19.615	**84.130**	98.430	1.570	51.875
5	Dice Loss (DL) [23]	α = 0.5, β = 0.5	1 × 10^−7^	ACU-Net (Ours)	**3.89**	**93.23**	20.06	**88.22**	**97.85**	**2.15**	**54.14**
				Vanilla U-Net	3.07	89.64	**23.29**	82.03	98.35	1.65	52.66
6	Focal Tversky Loss (FTL) [21]	α = 0.3, β = 0.7, γ = 0.75	1 × 10^−7^	ACU-Net (Ours)	**3.62**	89.31	**28.35**	81.43	**98.07**	**1.93**	**54.89**
				Vanilla U-Net	2.905	**90.865**	19.620	**84.125**	98.430	1.570	51.870

## Data Availability

The Mendeley PL dataset is available at http://dx.doi.org/10.17632/twxp8xccsw.9 (accessed on 28 January 2021) and the Powerline Dataset of Urban Scenes (PLDU) is available at https://drive.google.com/drive/folders/1XjoWvHm2I8Y4RV_i9gEd93ZP-KryjJlm(accessed on 17 December 2020).

## Supplementary Materials

Open-source implementation along with the reproducible results for all the experiments in this study is available at https://github.com/rubeea/focal_phi_loss_mmsegmentation (accessed on 20 January 2021).

## Abbreviations

The following abbreviations are used in this manuscript:

ACU-Net	Auxiliary Classifier U-Net
Bg	Background
BCE	Binary Cross Entropy
BBCE	Balanced Binary Cross Entropy
DL	Dice Loss
DSC	Dice Score
FDR	False Detection Rate
FL	Focal Loss
FN	False Negative
FP	False Positive
FPL	Focal Phi Loss
FTL	Focal Tversky Loss
GT	Ground Truth
LR	Learning Rate
PL	Power Line
PLDU	Power Line Dataset of Urban Scenes
RoI	Region of Interest
TN	True Negative
TNR	True Negative Rate
TP	True Positive
TPR	True Positive Rate
TL	Tversky Loss
UAV	Unmanned Aerial Vehicles
WBCE	Weighted Binary Cross Entropy

## Notations

The following notations are used in this manuscript:

A	Majority class
B	Minority class
(y,ŷ)	y as the actual probabilities and ŷ as the predicted probabilities
p_t_	Predicted probabilities in Focal Loss
L_BCE_	BCE Loss
L_WBCE_	Weighted BCE Loss
L_BBCE_	Balanced BCE Loss
L_FOCAL_	Focal Loss
L_DICE_	Dice Loss
L_TVERSKY_	Tversky Loss
L_MCC_	MCC Loss
α,β	Class-wise weights
γ F	ocal parameter
ε	Smoothing factor for loss functions
ρ	Class imbalance level
Ci	is a set of examples in class *i* for calculating *ρ*
S_α_	Smoothing parameter for exponential moving average
DSC_Bg_	Dice score for background class
DSC_PL_	Dice score for power line class
